# Supplementary material for: Theobromine Improves Working Memory by Activating the CaMKII/CREB/BDNF Pathway in Rats
Source: Nutrients. 2019 Apr 20;11(4):888. doi: 10.3390/nu11040888 (PMC6520707; doi:10.3390/nu11040888)
Supplement: Supplementary file 1 [file nutrients-11-00888-s001.zip › Supplementary file 2.docx]

**Supplementary file 2**


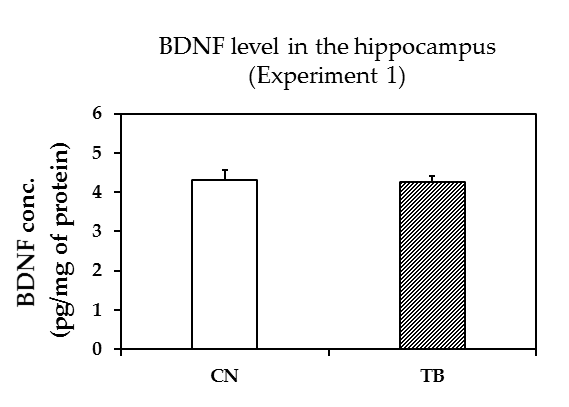


**Figure S1:** The effect of theobromine (TB) on the brain-derived neurotrophic factor (BDNF) protein level in the hippocampus. BDNF protein level in the hippocampus of TB-fed rats (TB rats) did not changed significantly than that of control rats (CN rats). Values are the mean ± S.E.M. (n=5 for each group).


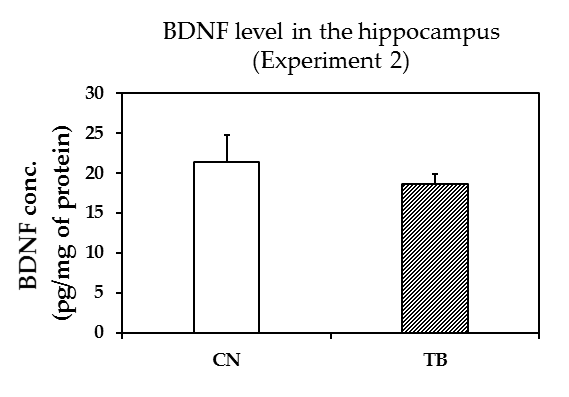


**Figure S2:** The effect of theobromine (TB) on the brain-derived neurotrophic factor (BDNF) protein level in the hippocampus. BDNF protein level in the hippocampus of TB-fed rats (TB rats) did not changed significantly than that of control rats (CN rats). Values are the mean ± S.E.M. (n=6 for each group).


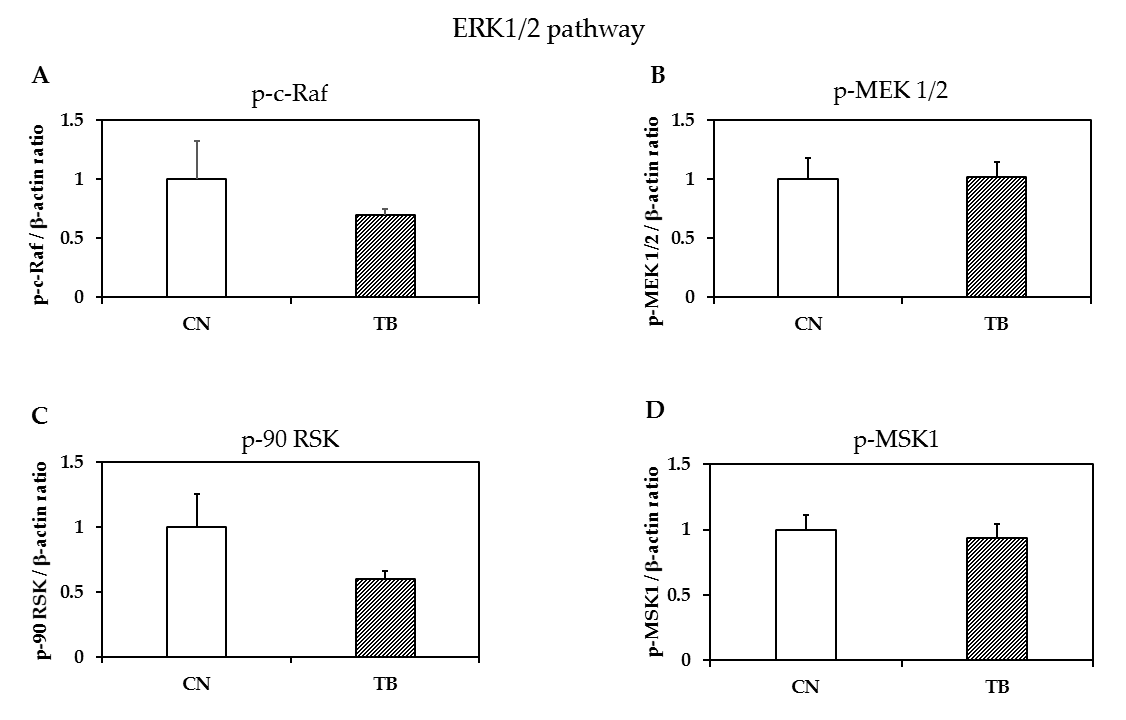


**Figure S3.** ERK1/2 pathway proteins levels in the medial prefrontal cortex (mPFC) of control rats (CN rats) and theobromine (TB)-fed rats (TB rats). TB did not changed (A) p-c-Raf, (B) p-MEK1/2, (C) p-90RSK; and (D) p-MSK1 levels in the mPFC. Values are the mean ± S.E.M. (n=6 per group).

**Table S2**: Correlation between biological markers and behavioural measurements

| **p-CREB** | **NOR Task (Discrimination index)** | | **Y-maze test (Percentage of Alteration )** |
| --- | --- | --- | --- |
|  | **Short term memory** | **Long term memory** |  |
|  |  |  |  |
| r - value | 0.468 | 0.480 | 0.700* |
| **p-CaMKII** | **NOR Task (Discrimination index)** | | **Y-maze test (Percentage of Alteration )** |
|  | **Short term memory** | **Long term memory** |  |
|  |  |  |  |
| r - value | 0.751* | 0.816** | 0.659* |
| **BDNF** | **NOR Task (Discrimination index)** | | **Y-maze test (Percentage of Alteration )** |
|  | **Short term memory** | **Long term memory** |  |
|  |  |  |  |
| r - value | 0.632* | 0.787** | 0.795** |

We have used 2nd trial’s results for novel object recognition (NOR) task and Y-maze test to measure the correlation with Biological marker phosphorylated CREB (p-CREB), phosphorylated CaMKII (p-CaMKII) and brain derived neurotropic factor (BDNF).

* Correlation is significant at the 0.05 level

** Correlation is significant at the 0.01 level
