# Supplementary material for: Theobromine Improves Working Memory by Activating the CaMKII/CREB/BDNF Pathway in Rats
Source: Nutrients. 2019 Apr 20;11(4):888. doi: 10.3390/nu11040888 (PMC6520707; doi:10.3390/nu11040888)
Supplement: Supplementary file 1 [file nutrients-11-00888-s001.zip › Supplimentary file 1.docx]

| **Primer name** | **Gen bank** | **Forward (5’ to 3’)** | **Reverse (3’ to 5’)** | **Products (bp)** | **References** |
| --- | --- | --- | --- | --- | --- |
| **BDNF** | **NM_001270630** | TAAATGAAGTTTATACAGTACAGTGGTTCTACA | AGTTGTGCGCAAATGACTGTTT | 88 | **https://doi.org/10.1093/cercor/bht203** |
| **β-actin** | **NM_031144** | TTGCTGACAGGATGCAGAA | ACCAATCCACACAGAGTACTT | 101 | **https://doi.org/10.1093/cercor/bht203** |

Supplementary file 1:

Table S1. Primer sequences for real time PCR
